# Cardiac Troponin: Fragments of the Future?

**DOI:** 10.1016/j.jacadv.2025.101695

**Published:** 2025-04-25

**Authors:** Samuel McGrath, Bashir Alaour, Thomas Kampourakis, Michael Marber

**Affiliations:** a King’s BHF Centre of Research Excellence, King’s College London, London, United Kingdom; b Division of Cardiovascular Medicine, University of Kentucky, Lexington, Kentucky, USA

**Keywords:** assay, fragments, myocardial injury, proteolysis, troponin

## Abstract

Cardiac troponin is the gold standard biomarker for the diagnosis of acute myocardial infarction (AMI). Development of high-sensitivity troponin platforms has revolutionized triage of chest pain patients, but specificity for type 1 AMI remains a clinical limitation. Consequently, differentiating type 1 AMI from other forms of myocardial injury is a common conundrum, heightened by the risks associated invasive coronary angiography. The troponin complex is a dynamic structure comprising of 3 subunits which variably fragment prior to measurement in the blood. Documenting the fragmentation patterns of cardiac troponin may help identify the cause of myocardial injury. This review explores the biology underlying troponin fragmentation and summarizes multiple lines of evidence that it can improve the specificity for diagnosis of type 1 AMI.

## Highlights

• Differentiating type 1 AMI from other forms of myocardial injury is a common clinical conundrum.
• The cTn complex fragments differently depending on myocardial injury, releasing various troponin species.
• Assays targeting or excluding specific troponin forms could enhance the diagnostic specificity for type 1 AMI.
• Further research is needed to identify circulating troponin fragments and develop novel high-sensitivity assays.

Cardiac troponin (cTn) is the worldwide gold standard biomarker of myocardial injury. Its elevation is essential for diagnosing acute myocardial infarction (AMI). The “Fourth Universal Definition of Myocardial Infarction” utilizes several criteria—including clinical presentation, electrocardiogram interpretation, elevation, and dynamic cTn levels—to class patients’ presentations into nonischemic myocardial injury or myocardial infarction (MI).^1^^,^^2^ The Fourth Universal Definition also defines multiple subtypes of MI depending on the underlying etiology. Differentiating AMI due to atherothrombosis (type 1) from another cause of myocardial supply-to-demand mismatch (type 2) is a common clinical conundrum and often requires invasive angiography.^3^

The evolution of high-sensitivity cTn (hs-cTn) assays has transformed chest pain pathways. Extremely low concentrations of cTn can now be detected in healthy individuals at low levels of imprecision.^4^^,^^5^ This has led to increased detection of abnormal troponin concentrations in the context of pathologies beyond type 1 AMI such as atrial fibrillation and sepsis.^6^ There has also been an acceptance of routine testing—even when the clinical likelihood of MI is low,^3^^,^^4^ thus compounding the problem further. Collectively, this has resulted in decreased specificity for type 1 AMI.

Ongoing research, as well as that over the last 2 decades, is examining the fragmentation pattern of cTn.^7^, ^8^, ^9^, ^10^, ^11^, ^12^, ^13^, ^14^ The ambition is that this may aid in the accurate diagnosis of different forms of myocardial injury, and possibly improve the specificity of type 1 AMI.^15^, ^16^, ^17^, ^18^, ^19^ The premise is the circulating species of cTn will be different depending on the underlying pathology, and thus the development of novel assays could target specific fragments. The purpose of this review is to explore the biology underlying troponin fragmentation and summarize the available evidence of potential clinical utility. We will start by briefly covering the nomenclature for the cTn complex used in this review.

## Troponin nomenclature

Troponin is the calcium-sensitive regulatory complex which comprises of 3 subunits: troponin C (TnC), the calcium-binding subunit; troponin I (TnI), the inhibitory subunit; and troponin T (TnT), which anchors the complex to the thin filament.^20^

For the purposes of this review, we refer to the following nomenclature throughout the article. T-I-C defines the full ternary troponin complex, I-C a binary complex of cTnI and cTnC, while cTnT, cTnI, or cTnC identify the individual monomeric subunits. Spans of contiguous amino acid residues within the primary structure of a monomer will be referred to as a region, and geographically adjacent areas of the ternary complex will be referred to as a motif. Thus, different regions of each monomer contribute to the motifs of the ternary complex, which then come together to make the functional machine of the T-I-C complex. Finally, the term epitope applies to either regions with a monomer or motifs (contributed by more than one monomer) that are recognized by an antibody.

A more detailed description of the cTn complex and its individual subunits can be found in the Supplemental Appendix.

## Troponin fragmentation

Proteolysis results in fragmentation of the cTn protein subunits, a process that occurs rapidly in the setting of cardiac necrosis as proteases are released from lysosomes. Another significant post-translational modification of cTn is phosphorylation, mediated by several protein kinases that modulate cTn structure and regulate cardiac function.^21^, ^22^, ^23^, ^24^, ^25^ While phosphorylation is an important mechanism, it lies outside the scope of this review. Phosphorylation and proteolysis also occur in other sarcomeric proteins such as cardiac myosin-binding protein C, but these processes are best understood for cTnI.

Proteins and their fragments can be detected using 2 principal approaches: physical separation or antibody-based identification.^26^ Commonly employed techniques include western blotting, which uses antibodies targeting cTn to detect specific fragments, and sandwich immunoassays that rely on at least 2 monoclonal antibodies targeting different regions of the troponin subunits, utilizing various technologies for signal production. Gel filtration chromatography is another method that separates molecules based on size and is often combined with sandwich immunoassays to identify troponin molecules in the collected fractions. Mass spectrometry, known for its precision, is also widely used and typically involves pre-enrichment of the target molecules through troponin-specific antibodies via immunoprecipitation or immunoaffinity chromatography.

In this section, we will focus on proteolysis of cTnI and cTnT subunits individually, followed by the implications of proteolysis on assay performance, specificity, and sensitivity.

### Cardiac troponin I

cTnI is very susceptible to proteolysis and will rapidly degrade when reconstituted into normal serum. It has been demonstrated that the folded central portion of the molecule (residues 39-134) is most resistant to proteolysis, but the largely unfolded flexible N- and C-terminal tails are highly susceptible regions.^7^

The calpain systems are intracellular calcium-dependent cysteine proteases which are activated by elevated intracellular calcium levels. The family consists of μ-calpain (calpain 1), μ--calpain (calpain 2), and calpastatin which inhibits the 2 calpains.^27^ Di Lisa et al demonstrated that cTnI is sensitive to μ-calpain cleavage. Furthermore, they showed that phosphorylation of the molecule alters the sensitivity to cleavage. Protein kinase A-mediated phosphorylation will protect against μ-calpain-mediated cleavage,^28^ whereas protein kinase C will have the opposite effect. M-Calpain has also been shown to cleave cTnI and mass spectrometry has highlighted multiple areas within cTnI’s N- and C-terminal extensions where this occurs.^29^

Matrix metalloproteinases (MMPs) are another protease family capable of cTnI cleavage, specifically MMP-2 which is the predominant cardiac isoform. MMPs are specifically activated in ischemia reperfusion injury as reactive oxygen-nitrogen species are produced.^30^ MMP-2 also cleaves the N/C terminal regions but has far fewer targets than calpains.^29^

**Figure 1:**
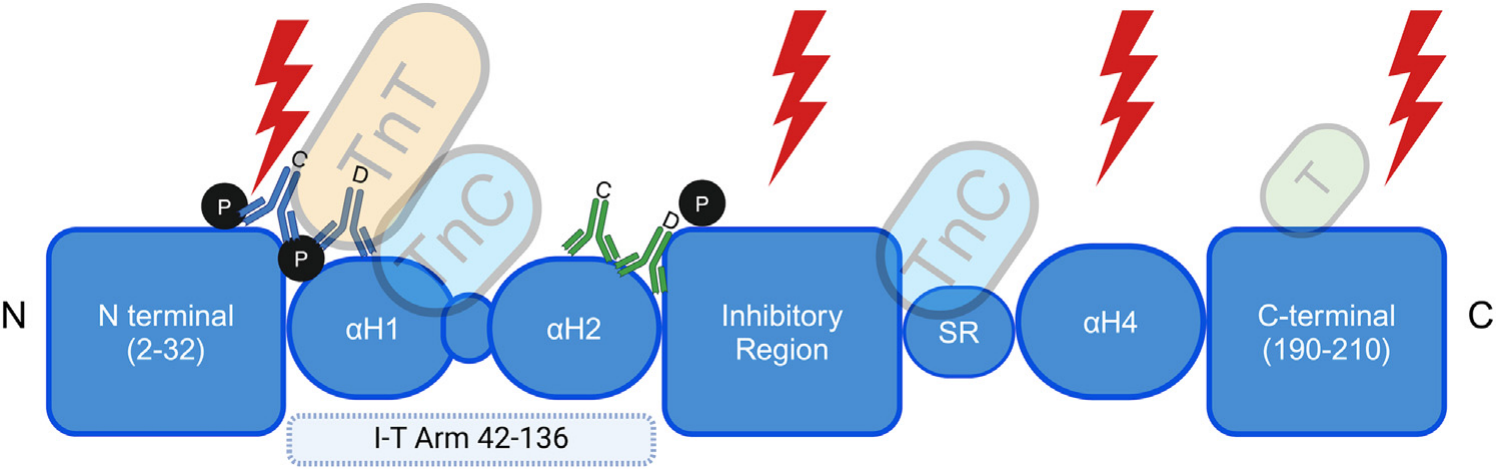
**Cardiac Troponin I** Cardiac troponin I is shown in blue with different functional regions labeled accordingly. Identified protease cleavage sites are marked with red lightning bolts and phosphorylation sites (P) are shown in black. The ovals indicate regions with helical secondary structure. SR indicates the switch region of cTnI. Known interaction sites with other subunits can be seen throughout the protein. The green box within the C-terminal region is where the cTnI complex interacts with tropomyosin-actin in the absence of calcium. The blue pair of antibodies represents the reagents of the Abbott Architect Assay, and the green pair represent the Roche E170 assay.^62^ The antibodies demonstrate the large differences between two commercial assays for cTnI. The annotations “C” and “D” above the antibodies denote capture and detection, respectively. cTnI = cardiac troponin I.

The cTnI protein and its sites of cleavage and phosphorylation are detailed in Figure 1.

### Cardiac troponin T

cTnT also has multiple cleavage sites which will differ in complexed and free forms.^15^ μ-Calpain causes intracellular proteolysis following AMI^28^^,^^31^ and like cTnI, the phosphorylation state will alter its sensitivity to cleavage.^28^ Caspases are cysteine-dependent proteases that cleave their targets after an aspartate residue (P1 position). They are activated in failing myocardium, induce apoptosis and the breakdown of myofibrillar proteins. Caspase-3 will target the N-terminal region of cTnT, but does not affect free cTnT.^32^ There are also several sites of relevance for proteolysis identified using mass spectrometry. The areas of interest include residues 54 to 94, residue 164, and multiple other sites between residues 189 to 223.^33^, ^34^, ^35^ The C-terminal portion of cTnT can also be cleaved, and this is thought to occur at Gln-199 and Lys-200. More recently, Katrukha et al identified 23 different cTnT fragments using a combination of western blotting and immunofluorescence assays. They identified residues 68 to 69 as a site of major proteolysis, as well as also identifying residues 190 to 223.^34^

Studies have shown that cTnT will degrade in serum samples, but remain predominantly intact in plasma samples,^36^ suggesting that the main cTnT degradation pathway is via plasma proteases. However, degradation in serum was presumed to be caused by μ-calpain despite this being an intracellular protease.^35^ Katrukha et al have shown that thrombin, a serine protease which is activated during coagulation, is capable of cleaving cTnT at R68/69 site of the N-terminus. This group hypothesized that thrombin is responsible for the breakdown of cTnT in serum samples as there is activation of the coagulation cascade.^37^ This study suggests thrombin is potentially another protease capable of cTnT cleavage post-AMI as it will be activated during clot formation, and those with AMI have been shown to generate increased amounts of thrombin.^38^

**Figure 2:**
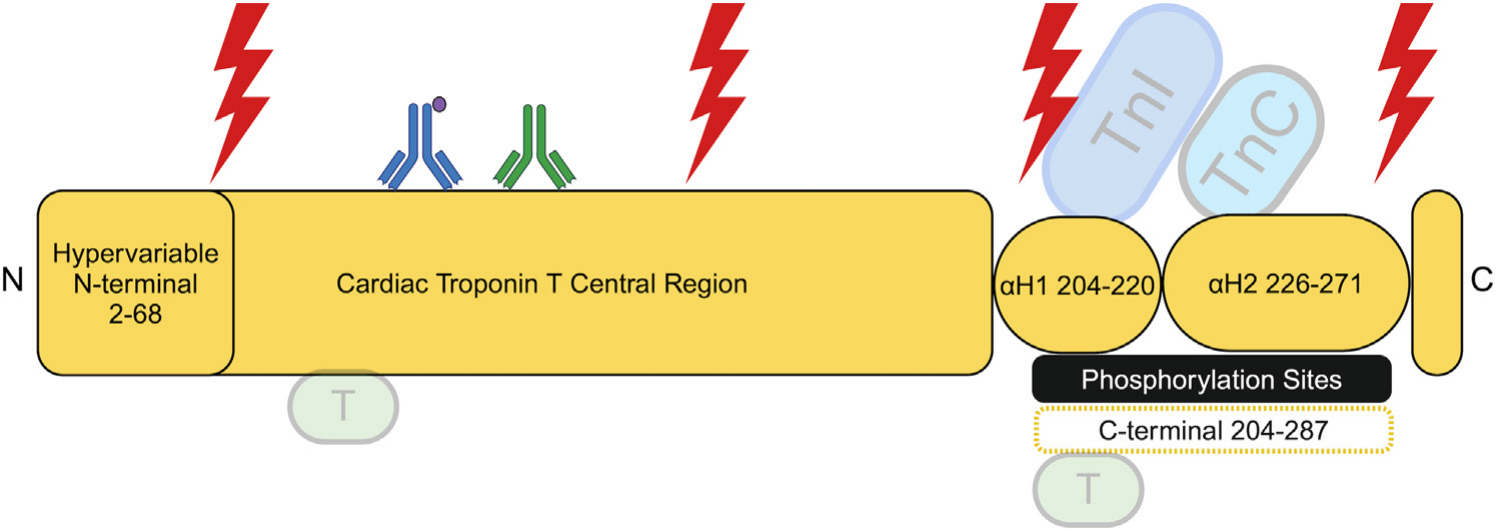
**Cardiac Troponin T** Cardiac troponin T is shown in yellow with different functional regions labeled accordingly. Cleavage sites are marked with the red lightning bolts and phosphorylation sites (P) are shown in black. The ovals correspond to the helical secondary structures in cTnT, and the interaction points with other troponin subunits can be seen within the C-terminus. There are also two sites of interaction with actin-tropomyosin which are indicated by green boxes. The position of the antibody pair used in the 5th generation Roche hs-cTnT assay are indicted accordingly. The capture antibody (blue) is directed against an epitope corresponding to amino acid residues 125 to 131 and biotinylated (purple) for immobilization. The detection antibody (green) is a ruthenium-tagged mouse-human chimera is directed against amino acid residues 136 to 147. Residues 203 to 271 of TnT interact with TnI and TnC and contribute to the I-T arm motif of the whole complex. cTnT = cardiac troponin T.

The cTnT protein and its sites of cleavage and phosphorylation are detailed in Figure 2.

### Implications of cTn fragmentation for assay development

Altering the conformation or length of individual cTn subunits has complex and unpredictable effects on the measurement of cTnI and cTnT by sandwich immunoassay. If either the capture or detection antibody targets an epitope that has been cleaved during proteolysis then that specific troponin complex will not be detected. If a cleavage event occurs between the capture and detection epitopes, it may not affect the affinity of the MAbs but would still prevent the protein from being detected. In an attempt to mitigate the aforementioned factors, it has been recommended that cTn assays should use antibodies which target neighboring epitopes within the central portion of the molecule, as this portion will be less affected by the above modifications.^39^^,^^40^ This is particularly pertinent to cTnI assays given it is a highly flexible subunit, and targeting the central portion should also reduce the lack of standardization among the various assays.

The choice of capture and detection antibody is critical, and in order to correctly select the ideal reagent, manufacturers need to decide what they want to detect. This could be a specific cTn subunit, or alternatively, they may want to try and detect the full ternary protein (T-I-C complex) which would involve using capture and detection antibodies targeting different subunits or epitopes created by adjacent subunits. A capture/detection pair may target epitopes of a free subunit that may be significantly modified when in a binary complex or whole subunit. Additionally, antibodies spanning cleavage sites will only detect longer forms of the subunit. The clinical utility of this approach will be covered later in this review.

## Circulating troponin species in different forms of myocardial injury

Understanding the release and fragmentation patterns of troponin post-myocardial injury has been an area of active research for decades. We know that the troponin complex fragments, but which is the dominant species in circulation? When does proteolysis occur, and does it occur in the heart or in the circulation? These are just a few of the questions that remain a source of debate, and if we improve our understanding, this could lead to novel diagnostics to identify specific forms of myocardial injury. There remains no consensus on the dominant forms of cTn in the circulation post-AMI, and different groups have presented different findings regarding the dominant species found for each subunit, reflecting the complexity of fragment detection and identification.

In the following section, we will outline some of the earlier studies, as well as more recent work looking at cTn fragmentation. The early work focused on characterizing the dominant species of cTn post-AMI. With advancements in assay technology, the more recent studies have been able to look at site-specific and time-dependent breakdown of the cTn subunit in far more detail. Following this, we will briefly cover cTn fragmentation in myocardial stunning.

### Troponin in the bloodstream: Fragments or whole subunits post-AMI?

Early work of Katrukha et al used several antibodies focusing on the cTnI subunit. They used a fluoroimmunoassay and ran samples in the presence and in the absence of ethylenediaminetetraacetic acid. Ethylenediaminetetraacetic acid removes divalent cations and will therefore decrease I-C association. Several of their assays had reduced affinity for cTnI when the I-C complex was introduced, and the addition of ethylenediaminetetraacetic acid improved cTnI assay performance. The authors concluded that the majority of cTnI in the bloodstream post-AMI is complexed, with only a small proportion of free cTnI present. This study is important as it demonstrates altering the ratio of monomer cTnI to I-C or T-I-C can have a significant effect on an individual assay performance. This study was not able to comment on cTnT as the antibodies were only targeting cTnI.^41^ In contrast, Wu et al used gel filtration and commercial cTn assays to examine cTnT and cTnI in 3 post-AMI patients. They had similar conclusions for cTnI as they found limited free cTnI in the samples and suggested the predominant form was the binary I-C complex. They also found some T-I-C and fragmented free cTnT, but the majority of cTnT was in its free monomeric form.^9^ Giuliani et al used multiple different immunoassays to conclude that the dominant form of cTnI is the binary I-C complex, with ternary T-I-C and free cTnI being seldom present.^8^ A further study by Bates et al used gel filtration chromatography to show that cTnI predominantly exists as a binary I-C, with lesser quantities of ternary T-I-C, and no free cTnI. They also found that the predominant form of cTnT was free cTnT monomer.^10^ Madsen et al looked specifically at the fragmentation of free cTnI in human serum post ST-segment elevation MI (STEMI). They were not able to visualize complexes of cTnI. They found that fragmentation of free cTnI subunit occurs as early as 90 minutes following onset of symptoms. More degradation products were present if the infarct was of larger size, or there was a delay to thrombolysis treatment.^11^ Taken together, the cumulative data from these early studies suggest that the predominant form of cTnI in the circulation is as binary I-C complex while for cTnT the monomer is predominant. This suggests that either the ternary troponin T-I-C complex once released into the circulation rapidly dissociates into I-C and cTnT, or that I-C and cTnT are separately released into the circulation.

In the early 2000s, the study by Labugger et al compared cTnI and cTnT species in serum post-AMI, and in donor serum where the subunits had been spiked in. Interestingly, they found differences in the fragmentation of both subunits between the 2 groups hypothesizing that a degree of fragmentation occurs within the diseased myocardium post-AMI.^12^ Further studies have shown cTnI degradation products from isolated myocardial tissues, further re-enforcing the fact that a degree of fragmentation occurs within the myocardium, prior to release into the blood stream.^13^^,^^42^

More recently, Vylegzhanina et al utilized cTn autoantibodies (TnAAbs) which have been shown to interfere with the detection of cTnI. They utilized multiple different troponin assays specific to cTnI and I-C to detect multiple forms of cTn. They ran these assays on donor plasma and a buffer with spiked complexes. They also had plasma samples from AMI patients which had been mixed with TnAAbs-containing plasma or TnAAbs-free plasma. They compared the signals of the different samples to study the recovery of cTnI and the inhibitory effect of the autoantibodies. They determined that autoantibodies only inhibited measurement of T-I-C complexes, and therefore their presence should not affect total cTnI concentration in the samples from patients in whom I-C and free cTnI are the prominent forms. Their results showed that there was in fact a reduction in total cTnI measured in samples early post-AMI with TnAAbs, thus hypothesizing that there is a mixture of T-I-C and I-C which changes over time. The TnAAbs-induced decrease in signal was almost negligible at 36 hours suggesting the ratio gradually changes to predominantly I-C over time.^43^

**Figure 3:**
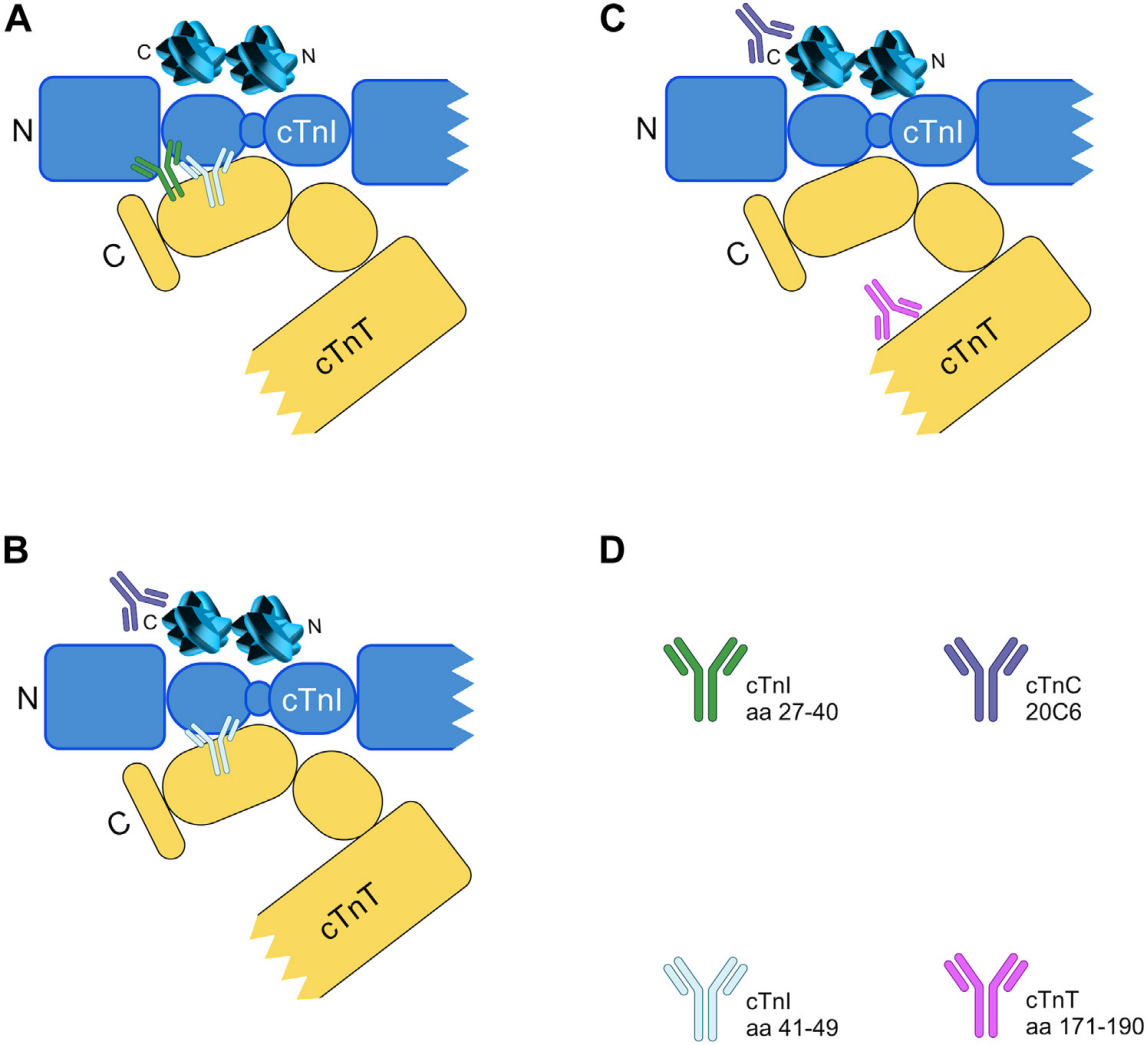
**Mixed Sandwich Immunoassays Targeting the Troponin Complex** This figure, adapted from damen et al,^47^ illustrates the mixed sandwich immunoassays used in their study. Similar approaches have been adopted by other groups using different antibodies targeting various epitopes of the individual subunits. In this figure, cTnT and cTnI are truncated at their N- and C-terminal ends, respectively. (A) Uses capture antibody to cTnI (aa 27-40) and detection to cTnI (aa 41-49). It will detect all forms of cTnI including T-I-C and non-complexed cTnI due to the close proximity of the antibodies. (B) Uses capture antibody 20C6, and detection antibody cTnI (aa 27-40). It cannot detect noncomplexed cTnI as the antibodies target different subunits. (C) Uses capture 20C6 and detection TNT1C11 (cTnT aa 171-190). It detects only full complex T-I-C or partially truncated T-I-C due to the location of the antibodies. (D) Details the antibodies used each assay. Abbreviations as in Figures 1 and 2.

In a separate study, the group spiked T-I-C, free cTnI, and binary I-C into plasma samples and used cTn assays utilizing multiple different antibodies to target different forms of the troponin complex. They labeled this a ‘mixed sandwich’ approach meaning the antibodies targeting different epitopes of cTnI/cTnT from different assays. This approach has also been utilized by other groups and is demonstrated in Figure 3. They demonstrated that the predominant form of troponin post STEMI within 10 hours of onset of symptoms is ternary T-I-C which compromises full-length or partially proteolyzed cTnI and a 29 kDa cTnT fragment. This complex subsequently fragments into binary I-C and free low-weight (15-20 kDa) central fragments of cTnT at 20 to 30 hours after symptom onset.^44^

Cardinaels et al showed similar findings for cTnT, demonstrating that circulating cTnT is highly degraded in the cases of AMI following rapid revascularization. Using a Roche Elecsys 2010 cTn assay and physical separation techniques, they examined fragments taken at multiple time points post-AMI. It was observed that the most abundant form of cTnT detected was also a mixture of cTnT fragments including the 29 kDa primary fragment as well as several secondary fragments. They also showed that the degradation of cTnT is dependent on time after symptom onset.^45^ However, they were unable to comment on the proportion of ternary T-I-C as the gel filtration technique did not provide adequate separation of the T-I-C complex from cTnT monomer. Streng et al determined that the 29 kDa primary fragment seen in many studies is an N-terminally cleaved degradation product of cTnT. The 16-kDa secondary fragment is further degraded at its C-terminus.^33^ Furthermore, Mingels et al showed that cTnT fragmentation pattern in end-stage renal failure (ESRF) is different from that in AMI. In patients with ESRF, the sera contain cTnT forms with truncated N- and C-terminal ends only (<18 kDa), suggesting that cTnT has completely degraded and it is the by-products/secondary truncated fragments that account for chronic troponin elevation seen in patients.^15^

From the more recent studies, we can conclude that the troponin complex will degrade in a time-dependent fashion. The studies described above have all taken blood at various time points post-AMI but have not examined the exact sites at which fragmentation occurs. Damen et al addressed this question by performing a pilot study on 2 patients from the TRAMICI (TRanscardiac Assessment of Myocardial Injury and Coronary Inflammation) study involving multisite sampling of cTn from the coronary venous and peripheral circulation at various time points post-AMI. Their group specifically looked at cTnT degradation and observed that cTnT is released in multiple forms from injured myocardium (ternary T-I-C, free TnT, and several TnT fragments) (52). Every cTnT form, including fragments (cTnT 29 kDa, 15-18 kDa) and free intact (cTnT 40 kDa), was detected in the coronary venous circulation. All forms had a higher concentration centrally than peripherally, and degradation occurred in a time-dependent fashion from symptom onset. In the early-presenting patient, the proportion of cTn in the T-I-C complex was still 25% (from peripheral artery), whereas in the late presenting patient, the T-I-C complex was almost absent (4%, peripheral artery).

They also found that fragments were detected in the coronary sinus, further re-enforcing the earlier studies hypothesis that cTn fragmentation could occur within ischemic cardiomyocytes. This study also confirms the findings of Katrukha et al that thrombin caused cTnT degradation,^37^ and therefore studies on cTn fragmentation should be carried out with lithium-heparin tubes for plasma samples rather than serum samples.

The group did a further study with a larger sample from the same cohort.^47^ This time they focused on cTnI and they used 3 different assays which were capable of detecting total cTnI, large size T-I-C, and complexed cTnI. The complexed cTnI assay could detect all formulations (binary/ternary, full size, and partially truncated) except free cTnI. Figure 3 displays their assays and these terms. This group showed that large size T-I-C and complex cTnI were more abundant in the coronary veins draining the infarct area than the peripheral circulation. Furthermore, there were marked differences within sites of the coronary venous circulation, again suggesting that degradation may not be exclusive to the peripheral circulation and that some degradation is likely to occur within necrotic cardiomyocytes. The proportions of complex cTnI/T-I-C to total cTnI remained relatively static between samples from the great coronary vein to coronary sinus in both early and late presenters, supporting the theory of intracellular cTn breakdown prior to release.

Katrukha et al demonstrated in vivo degradation of cTnT into multiple fragments.^34^ They also examined cTnT forms in vitro from samples taken post-AMI and separate samples with T-I-C spiked into normal plasma.^37^ With the exception of the C-terminus, they found no additional fragmentation of cTnT in incubated heparin-plasma samples. They have also demonstrated similar findings with cTnI.^48^ This group has also concluded that both subunits are stable in blood and hypothesized that the majority of the fragmentation occurs in the injured myocardium rather than in the circulation.

Advancements in assay development have increased sensitivity and enabled the identification of specific forms of the troponin complex. In combination, these studies allow us to draw the following conclusions.

• Troponin I and T undergo time-dependent fragmentation.
• It is likely the majority of fragmentation occurs in the circulation, with a lesser degree of fragmentation occurring in the myocardium prior to release.
• The exact mechanisms responsible for cTn proteolysis remain unclear.
• The cTn fragmentation patterns will depend on the time between blood draw and symptom onset, although less well characterized the etiology and severity of myocardial necrosis may also contribute.
• Presence of thrombin will cause cTnT fragmentation and therefore plasma may yield different results to serum.
• In patients with a shorter ischemic time prior to blood draw, there will be a higher proportion ternary T-I-C.
• Immediately after AMI, there will be a mixture of ternary T-I-C, binary I-C, and free TnT and all of these forms will fragment within the circulation in a time-dependent fashion.

### Troponin fragmentation in myocardial stunning

Myocardial stunning is the process of transient myocardial dysfunction following ischemia and subsequent reperfusion. Proteolysis of the cTnI subunit is also thought to play a possible role in this process. Isolated perfused rat hearts demonstrated a progressive degradation of cTnI following a period of ischemia, with fragments present in the stunned myocardium.^49^^,^^50^ The main fragment cTnI_1-193_ is missing 17 C-terminal residues. However, when cleavage was prevented global cardiac function improved following reperfusion.^49^ Murphy et al looked at this C-terminally cleaved cTnI fragment in more detail by creating transgenic mice expressing the modified cTnI_1-193_. All mice with this expression developed a phenotype of a stunned myocardium, with ventricular dilatation and worsening systolic and diastolic dysfunction.^51^ Isolated trabeculae from these mice have demonstrated worsening contractile reserve compared to controls,^52^ and it may have this effect by altering the calcium-sensing properties of the myofilaments. Feng et al subsequently demonstrated that cTnI proteolysis occurred in a Langendorff rat heart model with elevations in preload and stretch rather than ischemia, and inhibiting proteolysis in this model improved left ventricular contractile function.^53^

This remains an area of controversy as these cTnI findings did not translate in canine or porcine animal models.^54^, ^55^, ^56^ McDonough et al subsequently identified this fragment was present in the myocardium of bypass patients and hypothesized that this may still play a key role in myocardial stunning.^42^ These prior studies suggest that reversible myocardial dysfunction could be due to reversible cleavage of cTnI, but the precise mechanisms remain unclear.

## Understanding troponin fragments: Clinical relevance and future directions

### Clinical implications of fragment detection

There are 3 main limitations of current clinical cTn assays, the first is their inability to differentiate between early vs late presenters in AMI and the second is their lack of specificity for type 1 AMI. The final, and perhaps biggest, flaw is that they are unable to differentiate between different forms of myocardial injury. The ability to detect cTn fragments in different disease states potentially holds the key to solving these limitations, and exploratory work has been performed looking at cTn fragments in conditions other than MI.

Elevated cTn levels in patients with ESRF are common. Often, they are static concentrations above the sex-specific 99th percentile, and according to the universal fourth definition of MI, these patients would be labeled as chronic myocardial injury.^1^ Elevated troponin in this setting predicts increases in all-cause and cardiovascular mortality^57^^,^^58^ and the mechanisms responsible for the elevation are likely to be multifactorial. The clinical conundrum arises when these patients have atypical symptoms of chest pain or nonspecific electrocardiograms changes, and a clinician has to decide on the next steps in management.

Mingels et al looked into cTn forms in ESRF and showed that the circulating forms of cTn immediately post-AMI are different to those responsible for the chronic elevation in ESRF. Using western blot, they showed that the circulating fragments in ESRF are small N- and C-terminal truncated cTnT forms with a molecular weight of 18 kDa. This study also showed that when patients were dialyzed on a normal hemodialysis protocol, the fragments did not reduce, and neither did they change over 2 months,^15^ suggesting a constant fragmented cTn being consistently present. The same cTnT fragments were also shown to be present in blood samples taken from recreational runners after a marathon.^16^ The pattern of fragmentation and release kinetics in exercise are different to AMI. Clearly, the mechanism of troponin release in these 2 entities is different from necrotic cardiomyocytes decay in AMI. Understanding the release mechanism, and pattern restriction to small fragments under these 2 conditions may be of critical importance to understanding the concept of cTn release in reversible injury. Theoretically, knowledge from these studies could be used to make a more specific cTn assay for detecting AMI by excluding small cTn fragments from the analysis by using a combination of assays.

This concept was taken a step further by Airaksinen et al who used a novel immunoassay capable of detecting intact/mildly truncated cTnT which they termed “long” forms of cTnT and that excluded the short heavily truncated forms.^17^ They used the usual commercial Roche hs-cTnT assay to measure “total” cTnT and combined the assays to produce a ratio of long/total cTnT. The ratio of long/total was higher in non-ST-elevation myocardial infarction (NSTEMI) groups than in ESRF, with an area under the ROC curve of 0.955 (95% CI: 0.899-1.0) for discrimination between NSTEMI and ESRF if blood draws were taken within 24 hours of symptom onset. This is the first example of using multiple cTn assays to differentiate between forms of myocardial damage and provides proof-of-concept that knowledge of fragmentation can aid diagnosis. It provides an exciting insight into the potential of future commercial cTn assays and could improve our ability to triage and diagnose patients at the front door. The group has further optimized the sensitivity of their novel assay which now has an area under the curve of 0.986 for distinguishing the 2 conditions and has lowered the limit of detection by 28-fold.^19^ These 2 studies demonstrate the potential of novel assays in improving specificity for the diagnosis of AMI.

As this field progresses the pathophysiological dependence of fragmentation will become clearer but the reality is fragmentation is likely to be variable even within single disease entities. The studies above describe the combination of 2 assays, but as the field expands further assays targeting different fragments will evolve. Another strategy for clinical utility that we envisage is the combination use of multiple assays. Clinicians could order a “troponin panel,” thus allowing profiling of multiple fragments and their relative percentages. It is easy to envisage the clinical utility of this approach, for example, it could allow clinical profiling of NSTEMI patients at the front door, aiding triage for procedures. In the previous section, we discussed that T-I-C will be higher in the acute phase of an MI. If we were capable of examining the various cTn forms on the first presentation, this would give us an idea of the ischemic time window (early vs late), and potentially an indication of the severity of the injury. Zahran et al demonstrated the concept of combination assays in their pilot study utilizing 3 different troponin I assays to look at the serum of patients with raised cTnI. They found the profile of fragments between STEMI, NSTEMI, and type II MI was different, and with higher ischemic severity more proteolytic degradation of cTnI occurred.^59^

It is also possible that troponin fragmentation could distinguish between acute and chronic myocardial injury. Sörensen et al utilized 3 different commercial assays, 1 for cTnT and 2 for cTnI, to examine troponin complexes. The Atellica cTnI assay relies on antibodies which bind to more widely separated epitopes than the Architect cTnI assay, and thus may return lower values in chronic injury where there is degradation of the cTnI complex. This group showed that using these 2 assays the ratio of hs-cTnI to hs-cTnT was higher in acute injury, and lower in chronic injury, but the results did not support the hypothesis that the difference was due to degradation of cTnI since both cTnI assays returned similar concentrations. However, it does show that there is a difference in troponin subunit concentrations in different conditions which could have potential diagnostic benefit and should be researched further.^18^

Our Central Illustration highlights the possible clinical implications of combining knowledge of cTnT fragmentation in assay development. Assays can be developed that target (or exclude) portions of the troponin complex which are present in different forms of injury. Further work is needed in this field in characterizing fragments of the troponin subunits in different conditions and the timeline by which these processes occur. Nonetheless, this remains a promising area of research which may improve the specificity of cTn assays for AMI, as well as aiding clinician decision-making in other forms of myocardial injury.

**Central Illustration:**
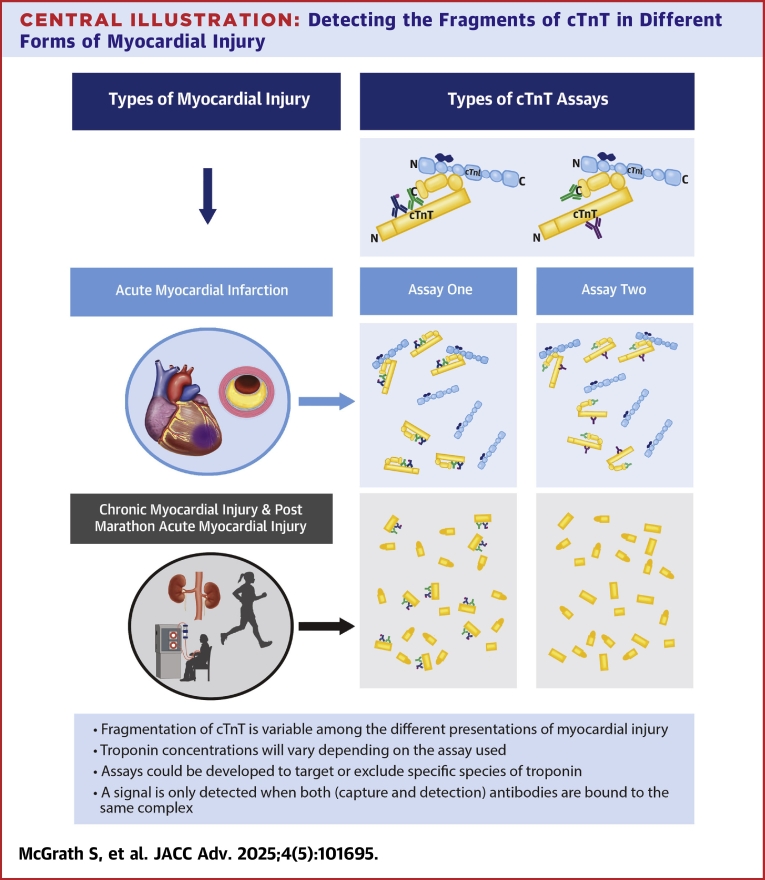
**Detecting the Fragments of cTnT in Different Forms of Myocardial Injury** cTnT fragmentation varies across different forms of myocardial injury, and overall ctnt concentration depends on the assay used. The blue circle represents acute myocardial infarction (AMI), where T-I-C fragments into free TnT and I-C. The yellow circle represents chronic myocardial injury, and postmarathon AMI. Here, cTnT appears as truncated N/C terminal forms and cTnI fragmentation is unknown. cTnT assay 1: A commercial hs-cTnT assay with a capture antibody targeting aa 125 to 131 and detection targeting aa 136 to 147, identifying intact and truncated forms of cTnT. cTnT assay 2: A central capture antibody and a C-terminal detection antibody, detecting longer cTnT forms while missing N/C-terminal fragments. This figure highlights variable fragmentation among types of myocardial injury. Troponin concentration depends on assay choice, as different assays target different portions of the complex. Assays could be developed to exclude specific fragments or target T-I-C to improve specificity for the targeted form of injury. Abbreviations as in Figures 1 and 2.

### The potential pitfalls and future directions of fragmentation work

A substantial amount of research into cTn fragmentation has occurred over the past 2 decades by leaders in the field, but despite this, clinical utility has remained predominantly speculative until recently. This may be a reflection of the intricacies of troponin and complexity of fragmentation, but nonetheless, there are several “known unknowns” that could be addressed in future work.

Firstly, several of the fragments will exist in the circulation at low individual concentrations, and thus the assays need to have exquisite sensitivity and low variability to be clinically useful. There have been huge improvements in assay development over the last decade, and the recent publication by Wittfooth et al of their novel assay picking up “long forms” of troponin has shown it is possible to develop these assays. Their assay utilizes up-conversion luminescence and performs with high sensitivity (limit of detection 0.4 ng/L, limit of quantification 1.79 ng/L),^19^ which is suitable for their cohort of interest. Future assays may need even higher sensitivity if looking to explore fragmentation in healthy individuals, and development of highly sensitive assays capable of dissecting the contribution of individual troponin forms to the total cTn signal, is certainly critical to advancing this field.

Degradation can occur in the myocardium, with further fragmentation in the bloodstream, and the potential for further fragmentation in vitro.^12^^,^^13^^,^^34^^,^^37^^,^^42^^,^^46^ There are several steps that will vary from patient to patient, and thus affect overall fragmentation patterns. For example, patients with longer presentation to revascularization times in STEMI will have more prolonged stasis within ischemic myocardium. In this instance, based on published data, one would predict more extensive fragmentation.

Katrukha et al’s work demonstrating cTnT fragmentation by thrombin in serum collection tubes highlights an in vitro variable that will affect interpretation of fragments.^37^ Further work is needed looking at preanalytic factors such as collection, storage, and handling. Interestingly, thrombin is also a potential confounding factor in vivo given its prevalence in ischemic heart disease.^60^

Understanding the mechanisms of release of cTn in different diseases is also critical, and further work is needed. As a recent editorial articulates, if apoptosis demonstrated by Canty et al is the mechanism of release of cTn fragments,^61^ then this mechanism is present in many cardiovascular diseases and could confound interpretation.^60^

Finally, while one hopes that each disease will have a distinct fragmentation pattern, the likelihood is an overlapping spectrum of patterns. The hope is that further dedicated research will resolve these patterns into distinct disease phenotypes.

## Conclusions

cTn is firmly established as the gold standard biomarker for myocardial injury, but the current clinical assays have been formulated to detect all circulating forms. The Fourth Universal Definition of MI clearly categorizes the pathophysiologies that result in cTn elevation but their identification in clinical practice is difficult. Even with ancillary imaging tests categorization often remains ambiguous. Advancements in cTn assay design allow us to measure low concentrations of cTn in the majority of the population and have also led to advancements in our knowledge of cTn fragmentation. Proteolysis of the cTn complex is sensitive to the pathophysiologic cause of myocardial injury but also changes over time. By developing assays to detect specific fragments, we may overcome the low specificity for type 1 AMI and potentially differentiate between forms of myocardial injury. Further work into troponin fragmentation is clearly required but has the potential to transform our practice and patient care in future.

## Funding support and author disclosures

Dr McGrath is undergoing a clinical research training fellowship (CRTF) funded by the 10.13039/501100000274British Heart Foundation (FS/CRTF/22/24187). Dr Marber is named as an inventor on a patent held by King’s College London for the detection of cardiac myosin-binding protein C as a biomarker of myocardial injury. All other authors have reported that they have no relationships relevant to the contents of this paper to disclose.
