# Supplementary material for: Cardiac Troponin: Fragments of the Future?
Source: JACC Adv. 2025 Apr 25;4(5):101695. doi: 10.1016/j.jacadv.2025.101695 (PMC12102503; doi:10.1016/j.jacadv.2025.101695)
Supplement: Supplemental_Appendix [file mmc1.pdf]

Supplemental Appendix

The Sarcomere and Troponin Complex

Thin- (actin) and thick- (myosin) filaments comprise the sarcomere which is the fundamental contractile unit of the heart. The thin filament is an allosteric system comprising of a double helix of filamentous-actin protomers (F-actin), and multiple regulatory protein subunits including tropomyosin and troponin. Calcium activation of the thin filaments triggers a conformational change in troponin and tropomyosin that uncovers myosin binding sites on actin, allowing myosin motors from the neighbouring thick filaments to bind and drive filament sliding and force generation [1–3].

Troponin is the calcium-sensitive regulatory complex within the thin filament, which is essential for the contraction of all striated muscles (fast/slow skeletal and cardiac). It is comprised of three sub-units: troponin C (TnC), the calcium-binding subunit; troponin I (TnI), the inhibitory subunit; and troponin T (TnT), which anchors the complex to the thin filament[4]. There are multiple isoforms of each troponin subunit, some of which are expressed only in cardiac muscle. TnC has a common isoform for cardiac and slow-skeletal muscles, and a separate isoform exclusively expressed in fast-skeletal muscles. In contrast, TnT and TnI have independent cardiac, and fast and slow skeletal muscle isoforms [1,3,5,6].

The whole Tn complex (T-I-C) has an elongated shape with a central core and several unstructured tails and linkers [7]. Takeda et al. describe the core structure of troponin as made up of two motifs. The I-T arm is comprised of a coiled coil formed from residues 42–136 of TnI and 203–271 of TnT, and is strongly bound to the C-terminal lobe of TnC (amino acid residues 93–161). The regulatory head comprises the N-terminal lobe of TnC (amino acid residues 1–

84), which can bind to troponin I in a calcium-dependent manner. Overall, the complex is dominated by alpha-helices joined by flexible linkers, this results in an asymmetric, highly flexible regulatory protein assembly which is essential for thin filament function [8]. The structure of the T-I-C complex is shown in **Supplemental Figure 1.**

Troponin I (TnI, 24kDa) is responsible for inhibiting acto-myosin ATPase activity in the absence of calcium**.** Troponin I consists of a cardiac-specific N-terminal extension, followed by a region that forms the I-T arm, followed by the inhibitory and switch region, and a C-terminal tail [8,9]. The N-terminal extension is specific to the cardiac isoform of TnI and interacts with the N-terminal lobe of TnC in a phosphorylation-dependent manner [10]. Moreover, there are other cardiac specific epitopes scattered throughout the entire cTnI subunit. The inhibitory and C-terminal region strongly bind to actin and tropomyosin, and block myosin binding to actin when no calcium is present [11]. Once activated by calcium, the N-terminal lobe of TnC binds the switch region of TnI, which leads to the detachment of the C-terminal region of TnI from actin-tropomyosin (Fig. 1). Subsequently, tropomyosin azimuthally moves around the thin filament, exposing myosin-binding sites on actin and triggering the formation of force-generating acto-myosin cross-bridges [8,9,12]. The C-terminus in particular, is critical to the inhibitory function of cTnI [13], and for stabilising the ternary troponin-actin-tropomyosin interaction in the absence of calcium [14].

Troponin T (TnT, 37kDa) anchors the troponin complex in the thin filament and crosslinks the two tropomyosin strands on opposite sides of the filament. It comprises of 288 amino acids, from which the middle residues 183-288 contribute to the core of the troponin complex (alongside the other subunits) [8]. The hypervariable N-terminus of cTnT (residues 1-68) differs between species and isoforms, and like cTnI it has a cardio-specific primary structure [7], with further cardiac specific epitopes scattered throughout the subunit. The cTnT molecule has two

tropomyosin-troponin binding sites (residues 89-127 and 215-240), and the C-terminal region of cTnT contains two alpha helices which interact with both cTnI and TnC [9].

Troponin C (TnC, 20kDa) is the calcium-binding subunit of the troponin complex and interacts with both cTnT and cTnI. TnC has a dumbbell-like structure with two lobes (N- and C-terminal) each formed from two EF-hand motifs. The C-terminal lobe exhibits two high affinity Mg^{2+}/Ca^{2+} binding sites (sites III and IV) that have a mainly structural function and help anchoring TnC in the troponin complex. In contrast, the N-terminal lobe has a single regulatory calcium binding site (site II), which controls the calcium-dependent interaction with the switch region of TnI [15]. Calcium binding site I is dysfunctional in cardiac troponin C but plays a regulatory role in fast skeletal TnC.

References

1. Gomes AV, Potter JD, Szczesna-Cordary D. The Role of Troponins in Muscle Contraction. IUBMB Life Int Union Biochem Mol Biol Life. 2002 Dec 1;54(6):323–33.

2. Tobacman LS. Thin Filament-Mediated Regulation of Cardiac Contraction. Annu Rev Physiol. 1996 Oct;58(1):447–81.

3. Park KC, Gaze DC, Collinson PO, Marber MS. Cardiac troponins: from myocardial infarction to chronic disease. Cardiovasc Res. 2017 Dec 1;113(14):1708–18.

4. Greaser ML, Gergely J. Purification and properties of the components from troponin. J Biol Chem. 1973 Mar 25;248(6):2125–33.

5. Farah CS, Reinach FC. The troponin complex and regulation of muscle contraction. FASEB J. 1995 Jun;9(9):755–67.

6. Gordon AM, Homsher E, Regnier M. Regulation of Contraction in Striated Muscle. Physiol Rev. 2000 Jan 4;80(2):853–924.

7. White SP, Cohen C, Phillips Jr GN. Structure of co-crystals of tropomyosin and troponin. Nature. 1987 Feb;325(6107):826–8.

8. Takeda S, Yamashita A, Maeda K, Maéda Y. Structure of the core domain of human cardiac troponin in the Ca2+-saturated form. Nature. 2003 Jul;424(6944):35–41.

9. Katrukha IA. Human cardiac troponin complex. Structure and functions. Biochem Mosc. 2013 Dec;78(13):1447–65.

10. Hwang PM, Cai F, Pineda-Sanabria SE, Corson DC, Sykes BD. The cardiac-specific N-terminal region of troponin I positions the regulatory domain of troponin C. Proc Natl Acad Sci. 2014 Oct 7;111(40):14412–7.

11. Kobayashi T, Patrick SE, Kobayashi M. Ala Scanning of the Inhibitory Region of Cardiac Troponin I. J Biol Chem. 2009 Jul;284(30):20052–60.

12. Li MX, Spyracopoulos L, Sykes BD. Binding of Cardiac Troponin-I $_{147-163}$ Induces a Structural Opening in Human Cardiac Troponin-C ˒. Biochemistry. 1999 Jun 1;38(26):8289–98.

13. Zhang Z, Akhter S, Mottl S, Jin JP. Calcium-regulated conformational change in the C-terminal end segment of troponin I and its binding to tropomyosin: Ca2+-regulated function of the C-terminus of TnI. FEBS J. 2011 Sep;278(18):3348–59.

14. Lehman W, Pavadai E, Rynkiewicz MJ. C-terminal troponin-I residues trap tropomyosin in the muscle thin filament blocked-state. Biochem Biophys Res Commun. 2021 Apr;551:27–32.

15. Collins JH. Myosin light chains and troponin C: Structural and evolutionary relationships revealed by amino acid sequence comparisons. J Muscle Res Cell Motil. 1991 Feb;12(1):3–25.

Supplemental Figure 1: Structure and interactions of the troponin complex

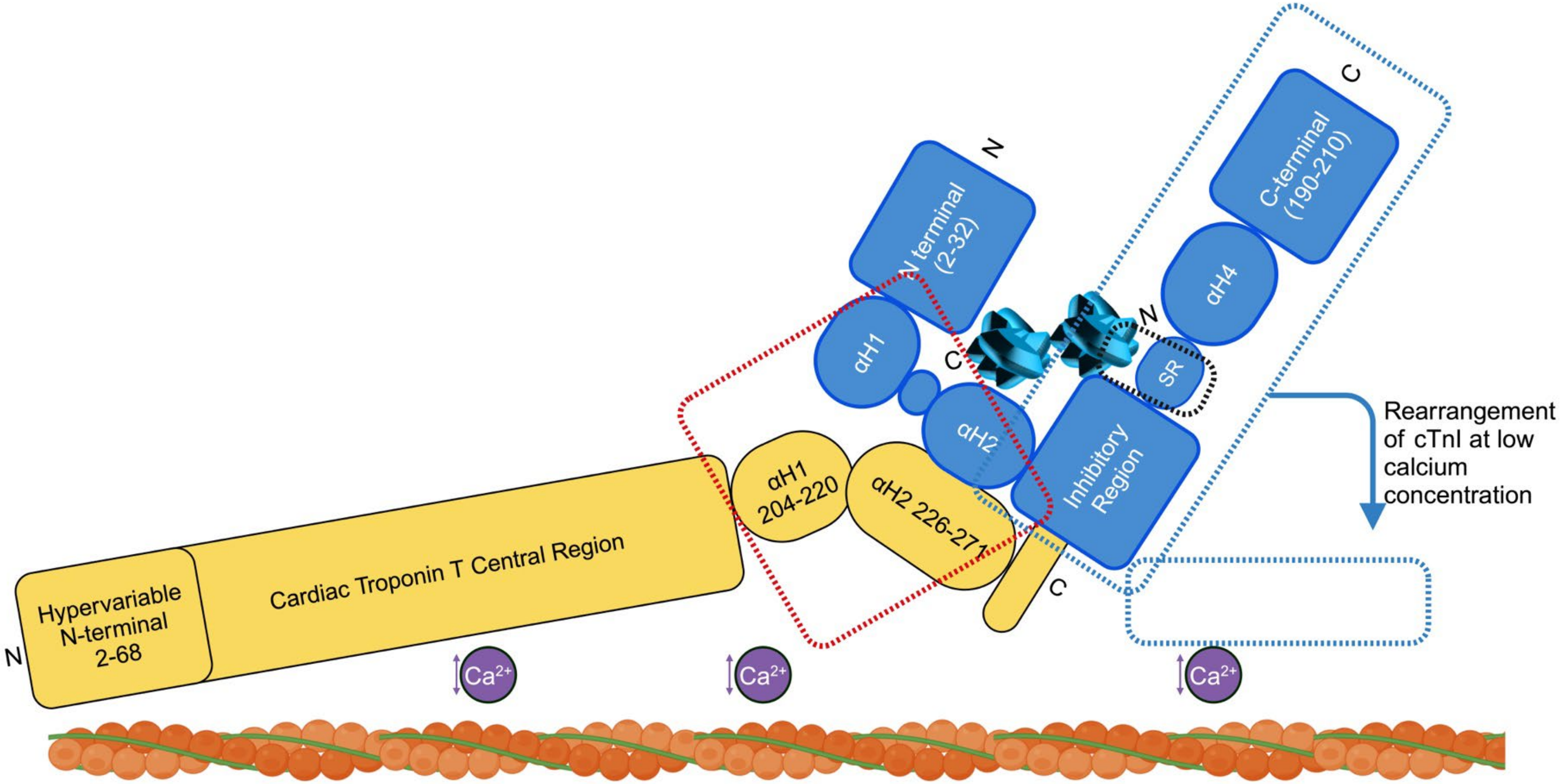

The ternary troponin (T-I-C) complex is shown above in the calcium-bound state (myosin binding site on actin exposed). There are three subunits of cTn, (i) cTnT is represented in orange, (ii) cTnI in dark blue, and (iii) cTnC is the two light-blue structures representing four EF hands. Individual

regions of each subunit contribute to motif of the whole troponin complex. The I-T arm of the whole complex is outlined by the dashed red box, and the regulatory motif is shown as the black box. These two motifs comprise of the core structure of the troponin complex. The inhibitory motif of cTnI is represented by the blue box and is shown in the active (non-inhibitory) conformation with the myosin-binding site of active uncovered. SR refers to the switch region of cTnI.

The complex's interactions with the thin filament proteins are shown by the purple arrows. The orange beads demonstrate actin, and the green lines are tropomyosin. When calcium is present, the C-terminus region of cTnI is detached from actin-tropomyosin and associated with cTnC as shown in the figure (see dashed blue box). When calcium levels fall, there is a conformational change shown by the blue arrow, and cTnI forms an extra attachment to actin-tropomyosin. The ovals of each subunit represent the alpha helixes. This figure is adapted from Takeda et al. [8]
